# Supplementary material for: Cocoa Extract Provides Protection against 6-OHDA Toxicity in SH-SY5Y Dopaminergic Neurons by Targeting PERK
Source: Biomedicines. 2022 Aug 18;10(8):2009. doi: 10.3390/biomedicines10082009 (PMC9405838; doi:10.3390/biomedicines10082009)
Supplement: Supplementary file 1 [file biomedicines-10-02009-s001.zip › biomedicines-1860666-SI.pdf]

Supplementary Materials: Supplementary Information

Table S1. Identification of phytochemical compounds in cocoa extract.

Peak	Tentative Identification	Molecular Formula	Ionization Mode	[M-H] (*m*/*z*)	MS/MS (*m*/*z*)	Error (ppm)
1	Theobromine	$C_7H_8N_4O_2$	$[M\text{-}H]^+$	181.0742	167.0102	0.80
2	Catechin-3-*O*-hexoside	$C_{21}H_{24}O_{11}$	$[M\text{-}H]^-$	451.1245	289.0710; 245.0818	−3.76
3	*N*-Caffeoyl-L-aspartate	$C_{13}H_{13}NO_7$	$[M\text{-}H]^-$	294.0619	179.0367; 276.0452	−2.82
4	Procyanidin B-type	$C_{30}H_{26}O_{12}$	$[M\text{-}H]^-$	577.1330	407.0740; 425.0839; 289.0688	0.87
5	Epicatechin	$C_{15}H_{14}O_6$	$[M\text{-}H]^-$	289.0706	245.0806; 205.0709	−3.81
6	Procyanidin B-type trimer	$C_{45}H_{38}O_{18}$	$[M\text{-}H]^-$	865.1984	577.1331; 407.0753; 289.0675	−0.23
7	Procyanidin B-type tetramer	$C_{60}H_{50}O_{24}$	$[M\text{-}2H]^{-2}$	576.1240	289.0719; 739.1666	−0.17
8	Procyanidin B-type tetramer (isomer)	$C_{60}H_{50}O_{24}$	$[M\text{-}2H]^{-2}$	576.1240	289.0719; 739.1666	−0.17
9	Procyanidin B-type pentamer	$C_{75}H_{62}O_{30}$	$[M\text{-}2H]^{-2}$	720.1569	289.06841; 407.0857	−0.95
10	Procyanidin B-type hexamer	$C_{79}H_{78}O_{44}$	$[M\text{-}2H]^{-2}$	864.1880	577.1302; 425.0808; 407.0757; 287.0537	1.85
11	Procyanidin A-type hexoside	$C_{36}H_{34}O_{17}$	$[M\text{-}H]^-$	737.1704	611.1376; 449.0839	−2.58
12	Quercetin 3-O-arabinoside	$C_{20}H_{18}O_{11}$	$[M\text{-}H]^-$	433.0733	301.0654; 271.8139	−2.93

Section S1. Quantitative Analysis

Theobromine was selected as external standard. Stock solution (1 mg/mL) was prepared in ethanol and the calibration curve was obtained in a concentration range of 10–200 µg/mL, with five concentration levels and triplicate injections of each level were run. Peak areas were plotted against corresponding concentrations. The amount of the theobromine in the sample was expressed as milligram per gram of extract.
